# The Rest

**Published:** 1877-10

**Authors:** 


### THE REST.

I AM dreaming of the blessings
  Just beyond the bounds of time,
Of the pearly-gated city,
  O'er whose wall no evils climb;
Where the Father folds his children
  Safely to his loving breast;
"Where the wicked cease from troubling,
  And the weary are at rest."

Now the toiling Christian pilgrim
  On a roughened pathway goes,
Here dejected, there disheartened,
  Ever harassed by his foes.
Pilgrim, raise thine eye above thee,
  There are joys for the oppressed,
"Where the wicked cease from troubling,
  And the weary are at rest."

Hast thou sickness, hast thou sorrow,
  Pains commingled with thy tears;
Canst thou trace the path of weeping
  Down the passage of the years?
"I am sick," none say in heaven,
  None by sorrow are possessed,
"Where the wicked cease from troubling,
  And the weary are at rest."

Oh! the joys of holy dying!
  From a holy life they come;
Constant toiling for the Master
  Yet will bring the servant home;
When he calls the tired pilgrim
  To the mansions of the blest,
"Where the wicked cease from troubling,
  And the weary are at rest."

